# Mass spectrometry imaging of adipose tissue lipidome maps lineage-specific metabolite profiles

**DOI:** 10.1016/j.jlr.2026.100982

**Published:** 2026-01-19

**Authors:** Christian Potts, Sylwia Stopka, Juan Aristizabal-Henao, Matthew D. Lynes

**Affiliations:** 1 Center for Molecular Medicine, MaineHealth Institute for Research, Scarborough, ME, USA; 2 BPGBio, Framingham, MA, USA; 3 Graduate School of Biomedical Sciences and Engineering, University of Maine, Orono, ME, USA; 4 School of Medicine, Tufts University, Boston, MA, USA

**Keywords:** adipose, lipidomics, metabolomics, Trpv1

Adipocyte subtypes may drive a heterogeneous distribution of lipids in adipose tissue. Adipocytes from the transient receptor potential cation channel subfamily V member 1 (Trpv1) lineage are one such subtype (1). We mapped subcutaneous white adipose from Trpv1^cre^mT/mG mice with matrix-assisted laser desorption/ionization (MALDI) coupled mass spectrometry (MS) (Fig. 1A). Total ion current identified specific peaks with signal-to-noise-ratios over 10 (Fig. 1B). Confocal microscopy identified specific m/z peaks associated with the Trpv1 lineage (Fig. 1C). We demonstrated that the lipidomic landscape of adipose tissue can be associated with specific cell types and used to define tissue structure.

**Fig. 1:**
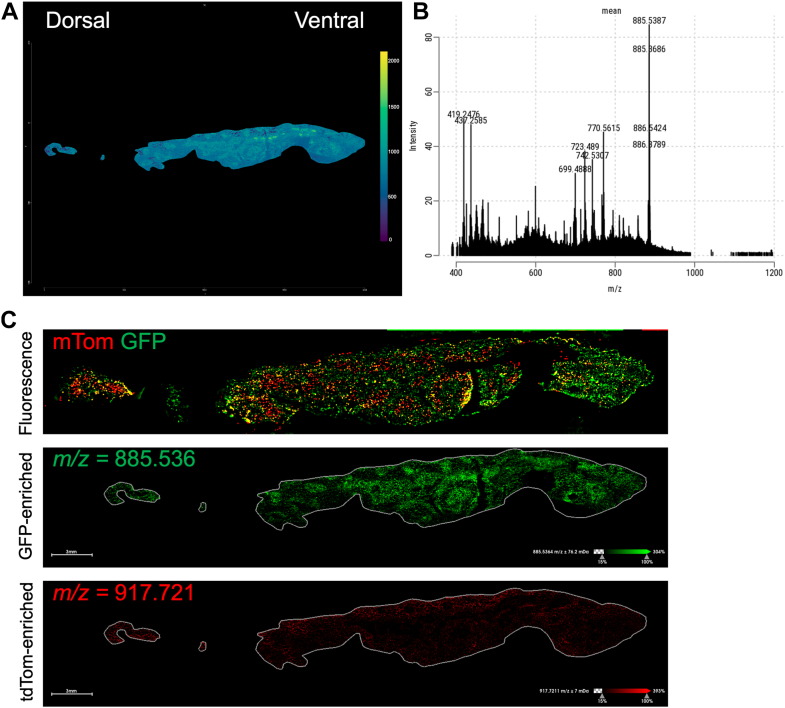
MALDI imaging of Trpv1^cre^mT/mG adipose tissue. (A) TIC image of inguinal subcutaneous adipose tissue from of Trpv1^cre^mT/mG mouse. (B) TIC histogram for of Trpv1^cre^mT/mG adipose tissue. (C) Fluorescence imaging of Trpv1^cre^mT/mG adipose tissue used to define regions for m/z peaks associated with the Trpv1 lineage.

**Equipment:** Bruker timsTOF flex.

**Data availability:** Source data and code are freely available at https://github.com/ChrPotts/SpatialMetabolomics_MHIR-BPGbio_2026.

## Conflict of interest

The authors declare the following competing interests: SS, JA-H, NRN, and MAK are and/or were employees of BPGbio Inc. The other author declares that they have no conflicts of interest with the contents of this article.
